# High-efficiency production of human serum albumin in the posterior silk glands of transgenic silkworms, *Bombyx mori* L

**DOI:** 10.1371/journal.pone.0191507

**Published:** 2018-01-19

**Authors:** Qiujie Qian, Zhengying You, Lupeng Ye, Jiaqian Che, Yiran Wang, Shaohua Wang, Boxiong Zhong

**Affiliations:** 1 College of Animal Sciences, Zhejiang University, Hangzhou, P. R. China; 2 Haining Sericulture Technology Extension Station of Zhejiang Province, Haining Zhejiang, China; 3 Department of Food Science and Nutrition, Fuli Institute of Food Science, Zhejiang Key Laboratory for Agro-Food Processing, Zhejiang University, Hangzhou, China; USDA Agricultural Research Service, UNITED STATES

## Abstract

Human serum albumin (HSA) is an important biological preparation with a variety of biological functions in clinical applications. In this study, the mRNA of a fusion transposase derived from the pESNT-PBase plasmid and a pBHSA plasmid containing the *HSA* gene under the control of a *fibroin light chain* (*FL*) promoter were co-injected into fertilized eggs. Fifty-six transgenic silkworm pedigrees expressing theexogenous recombinant HSA (rHSA) in the posterior silk glands (PSGs) with stable inheritance were successfully obtained. The SDS-PAGE and Western blot results confirmed that the rHSA was secreted into the transgenic silkworm cocoon, and the rHSA could be easily extracted with phosphate-buffered saline (PBS). In our research, the isolated highest amount rHSA constituted up to 29.1% of the total soluble protein of the cocoon shell, indicating that the transgenic silkworm produced an average of 17.4 μg/mg of rHSA in the cocoon shell. The production of soluble rHSA in the PSGs by means of generating transgenic silkworms is a novel approach, whereby a large amount of virus-free and functional HSA can be produced through the simple rearing of silkworms.

## Introduction

HSA is a non-glycosylated globulin, accounting for more than 50% of plasma proteins, and is the main factor contributing to blood colloid osmotic pressure. HSA can be used to transport drugs and metabolic factors because of its multiple binding sites [1,2], and is also an important biological product in modern medicine. It is widely used in blood volume expansion and human protein supplementation, especially in the treatment of shock due to blood loss, trauma and burns, in mitigating cerebral hypertension caused by brain edema and brain damage, and in preventing hypoalbuminemia, cirrhosis, and kidney disease [3]. Currently, the major commercial supply of HSA is derived from human plasma. This approach limits supply and increases the risk of virus infection, such as hepatitis and HIV [4,5].

rHSA has been produced in a methylotrophic yeast *Pichia pastoris* expression system and has shown no functional differences from plasma-derived HSA in clinical trials [6]. Mature HSA produced in transgenic rice seeds reached 10.58% of the total soluble protein in the rice grain and was equivalent to plasma-derived HSA in physical and biochemical characterization [7]. Other bioreactors that have been used to produce rHSA include *Kluyveromyces* yeast [8], mice mammary glands [9] and bovine blood [10].

The silk gland is divided into the anterior, middle and posterior silk glands and grows rapidly in fifth-instar larvae. Silk protein is mainly composed of 75% insoluble silk fibroin and 25% soluble sericin [11,12], which are synthesized by the posterior silk glands and the middle silk glands (MSGs), respectively. The fibroin is assembled from heavy chain (FH), light chain (FL) and P25 at the molar ratio 6:6:1[13,14]. The promoter of fibroin heavy chain or light chain has often been used in silk gland bioreactors [15,16,17,18,19]. The exogenous protein makes up to 15% in the most efficient silk gland bioreactor, which uses the fibroin heavy chain promoter [19]. However, the use of this promoter generates a chimeric protein in which the recombinant protein of interest is fused with silk fibroin. As a result, it is necessary to apply relatively harsh chemical methods to extract the foreign protein, a step that could potentially result in protein denaturation [20]. In recent years, some studies have expressed exogenous proteins in the MSGs, which require less stringent extraction methods for protein isolation. [21,22,23]. A recombinennt DsRed protein reached 9.5% (w/w) of cocoon shell weight using a modified sericin-1 expression systerm [24]. rHSA expressed using the sericin-1 promoter expression system achieved 3.0 μg/mg of the soluble protein in transgenic cocoon shells [23]. In addition, more than 50% of DsRed expressed using the P25 promoter was extracted from the cocoon shell using a moderate solution without dissolving fibrion [25]. We hypothesized that a soluble foreign protein expressed in PSGs could be extracted with only phosphate-buffered saline (PBS). In this study, we used the fibroin light chain promoter to express rHSA, and demonstrated via Western blot that it could be extracted with PBS from the transgenic cocoon shells. The isolated highest rHSA reached 29.1% of the soluble protein in the transgenic cocoon shell, which is equivalent to 17.4 μg rHsA per mg cocoon shell.

## Materials and methods

### Animals

*Lan* 10, a multivoltine non-diapause silkworm strain preserved in our laboratory, was used for transgenic experiments. Silkworm larvae were reared at 25°C with fresh mulberry leaves.

### The plasmid construction for expressing the rHSA gene

The plasmid pBHSA was constructed by cloning the 1758-bp mature HSA coding sequence amplified from the HSA cDNA vector preserved in our laboratory with the primers HSA-F and HSA-R (Table 1) into a pMD19-T vector. The plasmid p7801 containing the fibroin light chain promoter, the DsRed expression gene and *piggy*Bac transposon arms was constructed previously. pHSA and p7801 were digested by *EcoR*І and *BamH*I enzymes and ligated into pBH. The *fibroin light chain* signal peptide and His6 DNA sequence (FLSP-His6: *GGATCC*ATGAAGCCTATATTTTTGGTATTACTCGTCGTTACAAGCGCCTACGCTGCACCACATCATCATCATCATCATCCTCTAGA) were artificially synthesized and termed pFLSP-His6. pFLSP-His6 was cut with *BamH*I and *Xba*I enzymes and cloned into pBH to obtain pSBH. The 606-bp *fibroin light chain* PolyA signal sequence was amplified using PCR with flanking *Sal*I and *Hind*III enzyme sites (Table 1) and cloned into pSBH to acquire pSBHPA. The final plasmid pBHSA was digested and ligated using pSBHPA and p7801 with the *EcoR*I and *Afl*II enzymes.

**Table 1:** List of primer sequences used in this study.

Name	Sequence (5’-3’)
HSA-F^**a**^	**GATGATGATGATAAG**GATGCTCACAAGAGTGAGGT
HSA-R^**a**^	TTAGAGACCTAAGGCAGCTTGACTT
PA-F	GTCGACATAAGAACTGTAAATAATGTATATA
PA-R	AAGCTTCTTAAGGTGTGACTGCTTCGGACTACATTCT
R1-F	TCTGTATATCGAGGTTTATTTA
R1-R	CCGATAAAAACACATGC
R2-F	ACTCAAAATTTCTTCTAAAGTAACAA
R2-R	CTTTAACGTACGTCACAATATG
HSA-qF^**b**^	GGGGAGGTTTGGGTTGTCATCTT
HSA-qR^**b**^	ACCTATGGTGAAATGGCTGACTGC
Rp49-qF^**c**^	TGCTCCCAAATGGATTCCGTAAG
Rp49-qR^**c**^	CACGATCAGCTTCCGCTTCTTC

Note: a, an enterokinase cleavage site, DDDDK, (boldface) was incorporated in the forward primer HSA-F; b and c were used for qRT-PCR.

### Generation of transgenic silkworms

**Fig 1:**
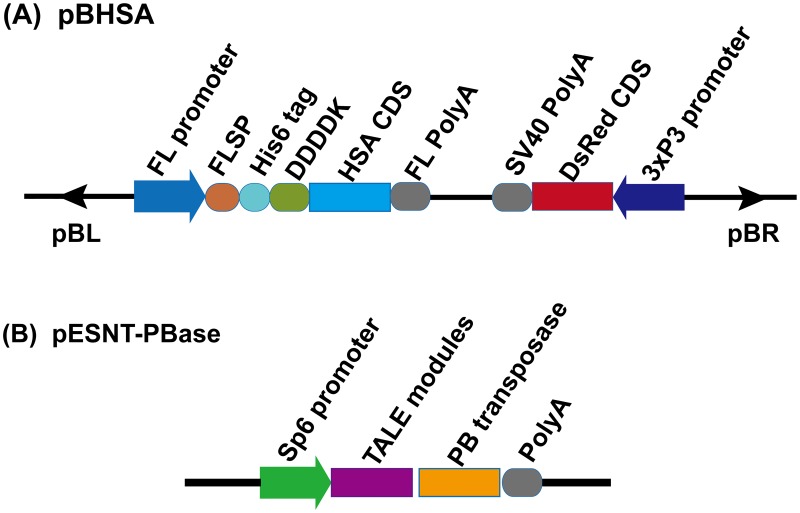
The structure of pBHSA and pESNT-PBase plasmids. **(A)**. Schematic representation of the pBHSA plasmid used in our transgenic experiment. pBL and pBR: the sequence of the left and right arms of the *piggy*Bac transposon plasmid; FL promoter, the promoter sequence of the *fibroin light chain* gene; FLSP, the signal peptide sequence of the *fibroin light chain* gene; His6 tag, the sequential 6×His-tag; DDDDK, enterokinase recognition site; HSA CDS, the HSA coding sequence; FL polyA, the polyA signal sequence of the *fibroin light chain* gene; 3×P3 promoter, the artificial promoter specifically driving the marker gene expression in the eyes and nervous system; DsRed CDS, the coding sequence of the red fluorescent protein gene; SV40 polyA, the SV40 polyA signal sequence. **(B)**. Schematic representation of the pESNT-PBase plasmid used in this transgenic experiment, which was constructed in our previous study [26].

The transgenic experimental method was performed as described in previous study [26] and is briefly described below. The mRNA of the fusion transposase pESNT-PBase was transcribed in *vitro* from the vector (Fig 1B) developed in our laboratory by using a mMESSAGE mMACHINE^®^SP6 transcription kit (Ambion, USA). The pBHSA plasmid (200 ng/μL) and the fusion transposase PESNT-PBase mRNA (200 ng/μL) were co-injected into eggs within 4 hours of oviposition. The eggs were incubated in a humidity chamber at 25°C. G0 moths were mated with wild-type *Lan* 10 to obtain the G1 generation. G1 eggs 6 to 8 days old were screened by fluorescence microscopy, and the individuals with red monocular eyes were transgene-positive silkworms.

### Detection of exogenous rHSA gene

Five transgene-positive silkworm pedigrees from the G1 generation were randomly selected. The genome DNA of the PSGs on 3-day fifth-instar larvae were extracted using a SanPrep Column Plasmid Mini-Preps Kit (Sangon Biotech, China). PCR was conducted on the transgene-positive silkworms to confirm exogenous gene expression using the HSA gene specific primer pairs: HSA-F and HSA-R (Table 1 and S1 Fig).

### Insertion site analysis

Inverse PCR was used to detect the insertion site. The genomic DNA extracted from the transgenic silkworm was digested with the restriction enzyme *Mbo*I and cyclized overnight at 16°C. The primers used in the first and second round of PCR were R1-F / R1-R and R2-F / R2-R (Table 1). The PCR product was cloned into a pMD19-T vector and sequenced by Sangon Biotech. The locations of the insertion sites were analyzed against the SilkDB database (http://silkworm.genomics.org.cn/silkdb/doc/release.html)

### Relative expression analysis of rHSA gene at transcript level using quantitative real-time PCR (qRT-PCR)

Quantitative real-time PCR experiments were performed to investigate the transcript level of the *rHSA* gene. Three 3-day fifth-instar individuals were randomly selected from the 5 transgene-positive silkworm pedigrees and the wild-type (WT) *Lan* 10 pedigree. The silkworms were dissected, and the PSGs were placed in DEPC-water containing 0.7% NaCl. Total RNA was extracted using Trizol after grinding in liquid nitrogen, and then reverse transcribed into cDNA by using the PrimrScript^®^RT reagent kit with gDNA Eraser (Takara, Japan). The relative expression of *rHSA* mRNA was quantified using a SYBR^®^Premix Ex TaqTMII kit (Takara, Japan) with HSA-qF and HSA-qR (Table 1) as the primers. The endogenous *B*. *mori Rp49* gene (*BmRp49*, accession number: NM_001098282) was used as a normalizer.

### SDS-PAGE and Western blotting analysis of rHSA protein in the transgenic cocoon shell

The protein samples were prepared as follows. First, 10 mg of cocoon shell was dissolved in 100 μL PBS buffer, ground in ice for 5 minutes, incubated at room temperature for 30 minutes, and centrifuged at 15000 rpm for 2 minutes at 4°C twice before the supernatant was extracted. The concentration was estimated in every protein sample by using a 2-D Quant Kit (GE company, USA), and the protein content in the cocoon was calculated. Each protein sample was diluted to a concentration of 2.5 μg/μl, added to the protein electrophoresis loading buffer (62.5 mM Tris-HCl, 2.5% SDS, 10% glycerinum, 0.5% bromophenol blue, 5% β-mercaptoethanol), and boiled for 5 minutes to denature the proteins. Then, 10 μl of the diluted sample was subjected to SDS-PAGE (12.5% polyacrylamide gel). The gel was stained with coomassie blue (0.1% Coomassie brilliant blue R-250, 10% acetic acid, 50% methanol). The content of rHSA was calculated by gray scale vale analysis [24] of coomassie blue stained gels using Gene Tools software. A separate 20 μl aliquot of the diluted sample was subjected to SDS-PAGE to perform Western blot analysis to verify the presence of the rHSA protein. The proteins were transferred onto a polyvinylidene difluoride (PVDF) membrane using a semi-dry transfer-blot at 1 mA/cm^2^ for 90 minutes. The PVDF membrane was washed and blocked in TBST (0.136 M NaCl, 20 mM Tris—HCl pH 7.6, 0.1% Tween 20) containing 3% bovine serum for 1 hour with gentle shaking at room temperature. Then, the membrane was incubated with a 10000-fold horseradish peroxidase (HRP)-conjugated His6 monoclonal antibody (Proteintech, China) for 1 hour at room temperature. After washing with TBST 3 times, the PVDF membrane was detected with an ECL luminescence reagent using a fluorescence and chemiluminescence imaging system (Gene Company, China).

### Purification of rHSA protein from the soluble protein of the transgenic cocoon shell

The rHSA protein was purified using an AKTA protein purification instrument (GE company, USA). The protein sample (5 ml) was added to a Ni^2+^ column. The rHSA protein was bound to the Ni^2+^ column with binding buffer (20 mM PBS, PH 8.0, containing 500 mM NaCl) and recovered with elution buffer (20 mM PBS, PH 8.0, containing 500 mM NaCl, 500 mM imidazole). The recovered solution was subjected to SDS-PAGE and silver staining.

## Results

### The design of rHSA plasmid

The rHSA plasmid, pBHSA, was constructed based on a *piggy*Bac transposon (Fig 1A). The DsRed gene driven by the 3×P3 promoter was chosen as the transgenic selectable marker, which was specifically expressed in the nervous system and eyes. The signal peptide of the *fibroin light chain* gene was designed to guide the rHSA protein secretion into the silk gland lumen from the PSG cells [27]. The His6 tag protein was used for easy recovery of the rHSA protein via a Ni^2+^ column. To harvest the mature human serum albumin, the enterokinase cleavage site (DDDDK) was added to the plasmid construction to remove the His6 tag.

### Screening of transgene-positive silkworms

**Fig 2:**
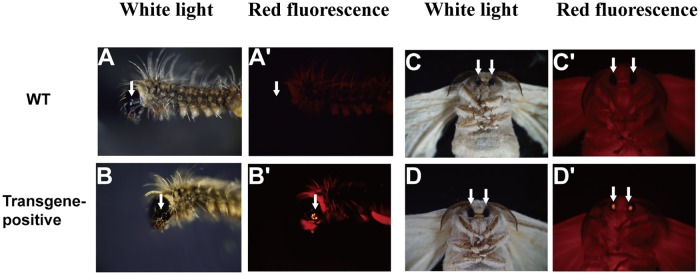
The fluorescence phenotypes of the DsRed-specific expressed in the eyes of the transgene-positive silkworm. **A** and **C** are the wild-type silkworm larvae and moths viewed under the normal light; **B** and **D** are transgene-positive silkworm larvae and moths viewed under the normal light. **A**' and **C**' are wild-type silkworm larvae and moths viewed under the red fluorescence; **B**' and **D**' are transgene-positive silkworm larvae and moths viewed under the red fluorescence. White arrows indicate the position of the eye.

Microinjected G0 generation silkworms were reared to mate with WT silkworm *Lan* 10 moths. G1 generation silkworms were screened by fluorescence microscopy (Olympus SZX16, Japan). The red monocular eyes and compound eyes could be observed in the transgene-positive silkworm larvae and moths, respectively, by fluorescence microscopy (Fig 2). In total, 56 pedigrees of the transgene-positive silkworm were selected for rearing, and the percentage of G1 positive brood was 54.4%. In our research, 5 pedigrees named HSA-1, HSA-2, HSA-3, HSA-4 and HSA-5, were selected for further examination. One silkworm from each of the 5 transgene-positive silkworm pedigrees and the wild-type *Lan* 10 pedigree was randomly selected to confirm the presence of the *HSA* gene in the transgene-positive silkworm. The genomic DNA of the PSG was extracted for PCR identification. The 1.8-kb DNA bands cloned from the transgene-positive silkworms were the same size as the *HSA* gene, which was subsequently sequence verified (S1 Fig).

### Insertion site analysis

**Fig 3:**
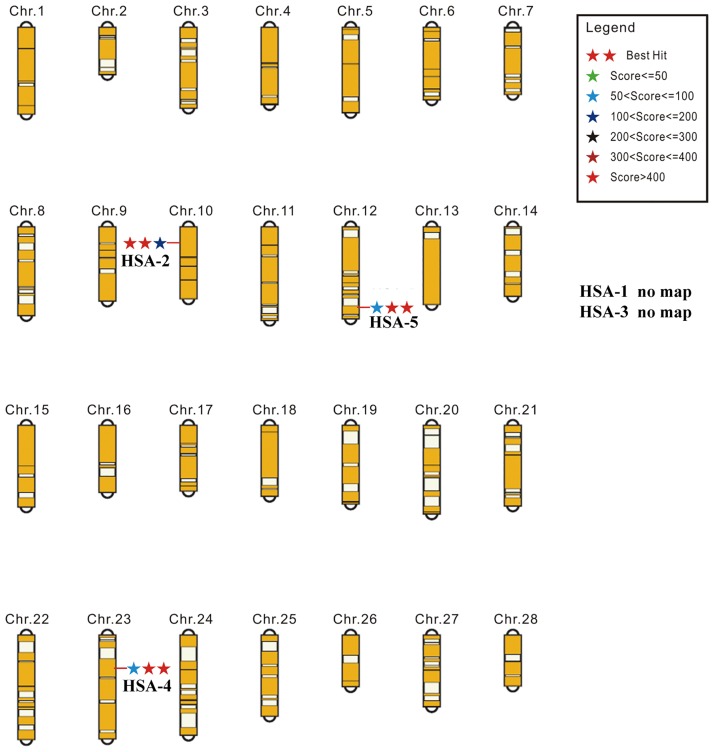
Locations of the insertion site of the transgenic silkworm pedigrees on the chromosome.

The *piggy*Bac transposon was randomly inserted into a TTAA four-base sequence. The locations of *piggy*Bac transposon insertion sites were scattered but were mainly in the intergenic and intron regions [28, 29]. We used the 5 transgene-positive silkworm pedigrees to conduct reverse PCR detection and to analyze the insertion sites. The foreign gene of the HSA-2 pedigree was inserted into intron regions, whereas the foreign gene of the HSA-4 and HSA-5 pedigrees was inserted into intergenic regions (Fig 3 and S1 Table). For the HSA-1 and HSA-3 pedigrees, we could not find a matching location based on the present genome database.

### Quantification analysis of rHSA gene expression among different transgenic silkworm pedigrees

**Fig 4:**
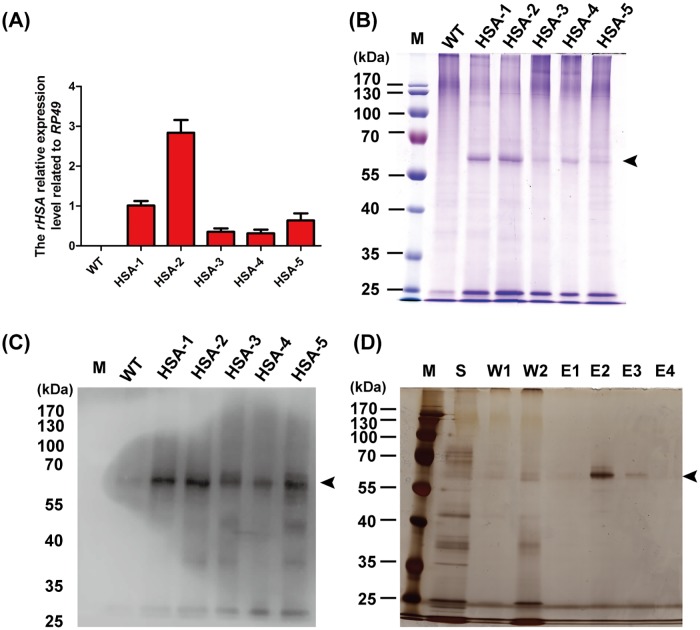
Identification of *rHSA* gene expression in transgenic silkworm pedigrees using qRT-PCR, SDS-PAGE and Western blot, and purification of rHSA protein from the cocoon shells. **(A)** The relative expression levels of the *rHSA* gene in the PSGs of the transgenic silkworm pedigrees on the 3^rd^ day of the 5^th^ instar were measured by qRT-PCR. WT: the wild-type silkworm *Lan* 10. HSA-1, HSA-2, HSA-3, HSA-4 and HSA-5: the transgene-positive silkworm pedigrees. (**B**) SDS-PAGE analysis of rHSA derived from the soluble protein of cocoon shells and (**C**) Western blot analysis of the cocoon layer. WT: the protein samples from the wild-type silkworm *Lan* 10. HSA-1, HSA-2, HSA-3, HSA-4 and HSA-5: the protein samples from the transgene-positive silkworm pedigrees. (**D**) The results of purification of the rHSA protein. S: the protein sample. W1, W2: recovered solutions after adding binding buffer. E1-E4: recovered solutions after adding elution buffer.

Position effects of gene expression have been revealed in *Drosophila* utilizing the *Cre/loxP* system, in which exogenous gene expression was more consistent when located in the same site and showed significant differences between different sites [30]. The *rHSA* genes of the 5 transgene-positive silkworm pedigrees were inserted into different sites of the genome. The mRNA level of *rHSA* was compared via qRT-PCR analysis. There was a large difference among the 5 transgene-positive silkworm pedigrees due to the different insertion sites. The mRNA expression level of *HSA-2* was the highest among the 5 pedigrees with a 9-fold increase in expression over the pedigree with the lowest expression, *HSA-4* (p≤0.01) (Fig 4A). These results suggested that exogenous gene expression was significantly affected by the insertion position.

### Analysis of rHSA protein in the cocoon shells of the transgenic silkworm pedigrees

The soluble proteins were extracted from the cocoon shell using PBS buffer. The SDS-PAGE results showed that the target bands with a molecular weight similar to that of rHSA were detected in transgene-positive silkworms but not wild-type silkworms (Fig 4B). The His6 antibody was used to further confirm the presence of the rHSA protein in cocoons (Fig 4C), and which showed that the transgenic silkworm could synthesize and secrete the rHSA protein from the silk gland cells into the PSG lumen and then spin it into a silk cocoon shell. rHSA is a soluble protein that can be extracted from the cocoon shell with PBS. Extracting and purifying the rHSA was feasible because of the simple composition of the cocoon proteins. The protein contents of HSA-1 and HSA-2 were the highest of the 5 transgene-positive silkworm pedigrees, which were consistent with the qRT-PCR analysis. The exogenous *rHSA* gene expression level of the mRNA and protein was highly consistent, which is also similar to that observed in our previous study [26]. The proportion of the rHSA protein was calculated by gray scanning. The human serum albumin of HSA-2 accounted for 29.1% of the soluble protein of the cocoon shells, meaning that a 1-gram cocoon shell contained 17.4 mg HSA protein (S2 Table).

### Purification of rHSA protein from the soluble protein of the transgenic cocoon shells

The recovered solution was silver-stained after SDS-PAGE electrophoresis. The protein samples extracted from the transgenic cocoon shells by PBS buffer contained various proteins. The cocoon proteins and the rHSA protein were in the recovered solutions after adding the binding buffer. The unique rHSA protein was concentrated in the recovered solutions after elution (Fig 4D), which indicated that the rHSA protein could be largely purified using a Ni^2+^ column.

## Discussion

We successfully generated transgenic silkworms expressing the foreign rHSA protein using elements of the fibroin light chain promoter and PolyA sequence. The foreign rHSA protein was synthesized and secreted in the PSG and spun into the cocoon shell. The rHSA protein, formed an average of 29.1% of the total soluble protein, and was harvested from the cocoon shell. The results suggested that the cocoon shell contained 17.4 μg rHSA/mg cocoon shell in the HSA-2 transgenic silkworm pedigree.

In previous studies, the exogenous DsRed protein expressed from the P25 promoter was distributed around the silk thread, rather than organizing into protein crystals. The exogenous protein thus might be physicalyl separated in the process of silk protein assembling, which could be beneficial to the extraction of exogenous proteins [25]. Indeed, the major royal jelly protein-1 expressed using the FL promoter could be extracted using PBS buffer from the cocoon layer [31]. In our research, the soluble globulin rHSA could likewise be extracted from the cocoon layer by PBS buffer. We assumed that a soluble exogenous protein expressed in the PSGs could be extracted using PBS buffer.

In a previous report, HSA was expressed in the sericin layer at 3.0 μg /mg cocoon [23]. However, the silk protein contained about 75% fibroin and 25% sericin. Therefore, the PSGs might be more robust for exogenous protein expression than the MSGs. Our research demonstrated that the utilization of the PSG bioreactor to produce the soluble exogenous protein could obtain higher yields than that of the MSG bioreactor.

The HSA protein is an important biological agent in the treatment of many diseases [3]. We have expressed HSA in transgenic silkworms by using a genetically engineered *piggy*Bac transposon system. The high expression and genetic stability of the transgenic silkworm are conducive to the development and application of rHSA.

## Supporting information

S1 Data Sequence of pBHSA transgenic plasmid used in this study. (DOCX)

S1 Fig The results of transgenic silkworm identification by PCR. (TIF)

S1 Table Genomic insertion sites of the pBHSA plasmid in 5 transgenic silkworm pedigree. (XLSX)

S2 Table The content rHSA protein in the cocoon shells from the HSA-2 transgenic silkworm pedigree. (DOCX)
