# Supplementary material for: High-efficiency production of human serum albumin in the posterior silk glands of transgenic silkworms, Bombyx mori L
Source: PLoS One. 2018 Jan 19;13(1):e0191507. doi: 10.1371/journal.pone.0191507 (PMC5774803; doi:10.1371/journal.pone.0191507)
Supplement: S1 Data — (DOCX) [file pone.0191507.s001.docx]

**S1. Data. The sequence of pBHSA** **transgenic plasmid used in this study.**

**> pBHSA transgenic plasmid (complete sequence)**

GATCTGAGCTCGCCCGGGGATCTAATTCAATTAGAGACTAATTCAATTAGAGCTAATTCAATTAGGATCCAAGCTTATCGATTTCGAACCCTCGACCGCCGGAGTATAAATAGAGGCGCTTCGTCTACGGAGCGACAATTCAATTCAAACAAGCAAAGTGAACACGTCGCTAAGCGAAAGCTAAGCAAATAAACAAGCGCAGCTGAACAAGCTAAACAATCGGGGTACCGCTAGAGTCGACGGTACCGCGGGCCCGGGATCCACCGGTCGCCACCATGGTGCGCTCCTCCAAGAACGTCATCAAGGAGTTCATGCGCTTCAAGGTGCGCATGGAGGGCACCGTGAACGGCCACGAGTTCGAGATCGAGGGCGAGGGCGAGGGCCGCCCCTACGAGGGCCACAACACCGTGAAGCTGAAGGTGACCAAGGGCGGCCCCCTGCCCTTCGCCTGGGACATCCTGTCCCCCCAGTTCCAGTACGGCTCCAAGGTGTACGTGAAGCACCCCGCCGACATCCCCGACTACAAGAAGCTGTCCTTCCCCGAGGGCTTCAAGTGGGAGCGCGTGATGAACTTCGAGGACGGCGGCGTGGTGACCGTGACCCAAGACTCCTCCCTGCAGGACGGCTGCTTCATCTACAAGGTGAAGTTCATCGGCGTGAACTTCCCCTCCGACGGCCCCGTAATGCAGAAGAAGACCATGGGCTGGGAGGCCTCCACCGAGCGCCTGTACCCCCGCGACGGCGTGCTGAAGGGCGAGATCCACAAGGCCCTGAAGCTGAAGGACGGCGGCCACTACCTGGTGGAGTTCAAGTCCATCTACATGGCCAAGAAGCCCGTGCAGCTGCCCGGCTACTACTACGTGGACTCCAAGCTGGACATCACCTCCCACAACGAGGACTACACCATCGTGGAGCAGTACGAGCGCACCGAGGGCCGCCACCACCTGTTCCTGTAGCGGCCGCGACTCTAGATCATAATCAGCCATACCACATTTGTAGAGGTTTTACTTGCTTTAAAAAACCTCCCACACCTCCCCCTGAACCTGAAACATAAAATGAATGCAATTGTTGTTGTTAACTTGTTTATTGCAGCTTATAATGGTTACAAATAAAGCAATAGCATCACAAATTTCACAAATAAAGCATTTTTTTCACTGCATTCTAGTTGTGGTTTGTCCAAACTCATCAATGTATCTTAAAGCTTATCGATACGCGTACGGCGCGCCAAGCTTAAGGTGTGACTGCTTCGGACTACATTCTCGTTATAGGGGCTTTATTAAAGTGAATTGTGCCTAATGCGATGAAAAAATAAGGTACATTTTTGTATGTTTCGCAGCTTAATCTCCGAATACATGAAAAAAATCGCATACAGTCTCTCCAGTTGGAAAGCAATTGACAACAGCACCGAAATCCATTGTATATGCCGTATGGTATATGGTATTATCGAATACCGTACTTATTTTAATGGGGCCAGAAATAACAAACTAAATTCTTTATCTGGAAAACTGGATACATCTTTATTGTACAAAAAAATCAATTAGAAGAACAATAAGATTGATCATCACCAAAATGAAAAGCCGTGTCGTTGATTTATATAATATATATTATATTAACGTATATTTATTTATATATTCATAAATAATCTAAAAAACTTCCAAAAAAAATCCTAACGCAAACACAATTTGCATAAAATGTCCTAAACACAATTAAAGTATACATAATTATTAATTTTAGTCTGTTTTTATATAGGTAGATTGTCTTATAATGATATATATATGTTTGTATATGGTTTATATATATCTTTTATATAATTATATATATACATTATTTACAGTTCTTATGTCGACGATTTTAGAGACCTAAGGCAGCTTGACTTGCAGCAACAAGTTTTTTACCCTCCTCGGCAAAGCAGGTCTCCTTATCGTCAGCCTTGCAGCACTTCTCTACAAAAGCTGCGAAATCATCCATAACAGCTTTCAGTTGCTCTTTTGTTGCCTTGGGCTTGTGTTTCACGAGTTCAACAAGTGCAGTTTGTTTCTTGATTTGTCTCTCCTTCTCAGAAAGTGTGCATATATCTGCATGGAAGGTGAATGTTTCAGCATTAAACTCTTTGGGAACGTATGTTTCATCGACTTCCAGAGCTGAAAAGCATGGTCGCCTGTTCACCAAGGATTCTGTGCAGCATTTGGTGACTCTGTCACTTACTGGCGTTTTCTCATGCAACACACATAACTGGTTCAGGACCACGGATAGATAGTCTTCTGCACAGGGCATTCTTTTTGCTTCAGGATGTTTACAACATTTGCTGCCCACTTTTCCTAGGTTTCTTGAGACCTCTACAAGAGTTGGAGTTGACACTTGGGGTACTTTCTTGGTGTAACGAACTAATAGTGCATTCTGGAATTTGTACTCTCCAAGCTGCTCAAAAAGCTCACAATTTTGTTTGATTAAATTCTGAGGCTCTTCCACAAGAGGTTTAAATTCATCGAACACTTTAGCATAGCATTCATGAGGATCTGCAGCGGCACAGCACTTCTCAAGAGTGGTTTCATATGTCTTGGCAAGTCTCAGCAGCAGCACGACAGAGTAATCAGGATGCCTTCTTGCATATTCATACAAAAACATGCCCAGGAAGACATCCTTTGCCTCAGCATAGTTTTTGCAAACATCCTTACTTTCAACAAAATCAGCAGCTAATGAAGGCAAGTCAGCAGGCATCTCATCATTTTCCACTTCGGCAATGCAGTGGGATTTTTCCAACAGAGGTTTTTCACAGCATTCCTTCAGTTTACTGGAGATCGAATCTTGATTTTCACAGATATACTTAGCAAGGTCTGCCCTGTCATCAGCACATTCAAGCAGATCTCCATGGCAGCATTCCGTGTGGACTTTGGTAAGATCTGTCACTAACTTGGAAACTTCTGCAAACTCAGCTTTGGGAAATCTCTGGCTCAGGCGAGCTACTGCCCATGCTTTGAAAGCTCTTTCTCCAAATTTTTGGAGACTTGCACACTTGAGTCTCTGTTTGGCAGACGAAGCCTTACCTTCATCCCGAAGTTCATCGAGCTTTGGCAACAGGCAGGCAGCTTTATCAGCAGCTTGGCAACATTCTGTAAAAGCAGCTTTATACCTTTTAGCAAAGAAAAGGAGTTCCGGAGCATAAAAGTAAGGATGTCTTCTGGCAATTTCATATAAGTATTTTTTCAAAAATGTCTCTTCATTGTCATGAAAAGCAGTGCACATCACATCAACCTCTGGTCTCACCAATCGGGGGAGGTTTGGGTTGTCATCTTTGTGTTGCAAGAAGCATTCATTTCTCTCAGGTTCTTGTTTTGCACAGCAGTCAGCCATTTCACCATAGGTTTCACGAAGAGTTGCAACTGTGCATAATTTGTCTCCAAAAAGGGTATGAAGTGATTTGTCACAATTTTCAGCTGACTCATCAGCAACACATGTTTTTGCAAATTCAGTTACTTCATTCACTAATTTTACATGATCTTCAAATGGACACTGCTGAAGATACTGAGCAAAGGCAATCAACACCAATGCTTTGAAATTTTCTTCTCCCAAATCTTTAAACCGATGAGCAACCTCACTCTTGTGAGCATCCTTATCATCATCATCAATCTCTAGAGGATGATGATGATGATGATGTGGTGCAGCGTAGGCGCTTGTAACGACGAGTAATACCAAAAATATAGGCTTCATGGATCCTTTAGTGGTCTGTTATGTGACCAATCGGTATATACTATACAGGATTCCGTGATTAATGTTCCACTATTTATATATAAAAAGACAATTAAAATAAATATCAAACAACTGTACCAACGAACGCTGACAGCATCTAACGTTCAAAAGATTTTGCATTTCAGTTAAAATTGACTATGCTTTATTATTGCGATTTTAAGGTTTAGTTTCTAATGAAGATGATTTCTTGAGGTGTACGCCTCTAGTTAAACTAAGTGTTGCTCGAAACAAAGCGATCTAATTGATCCGATTGTTCTTATATTTGTTCGCATCGAGAATTAACGCAAATTTAACTGTTGAATATGTAACAATGTGGAATTAAAATGTTTTGTAGTGTTAACGTCGTCTGTTATGTCAATGTCGGTAAATGACATTTTAAATAATTCATATAATTATTCTATGCATGTTTTTTTTTTTAATTTAAATTATTGAAGTTATACTTCTTTAGGCACGTTATGAAAAATCTATGAGAGTGAAATTTTACGATGCGCGCGCACCGTGACACAAAATTAACAGAATGAAGTTGCCCACTAAATCGCTCATTACAATACGACTGACGTAACTTGGCGAGTCTGAATATAGCCGCAGGTGCACTTTCCGAACCGTACCGAGAATTACCGAATTTATACGATGTCAAGAAAAGGGATGTCCAATATGCGCTCGAGCTGAGCAGAGAGGATATGCTCATCGTCTAAAGAACATCCCATTTTATTATATATTAGTCACGATATCTATAACAAGAAAATATATATATAATAAGTTATCACGTAAGTAGAACATGAAATAACAATATTAATTATCGTATGAGTTAAATCTTAAAAGTCACGTAAAAGATAATCATGCGTCATTTTGACTCACGCGGTCGTTATAGTTCAAAATCAGTGACACTTACCGCATTGACAAGCACGCCTCAGCCGAGCTCCAAGCGGCGACTGAGATGTCCTAAATTGCAAACAGCGACGGATTCGCGCTATTTAGAAAGAGAGAGCAATATTTCAAGAATGCATGCGTCAATTTTACGCAGACTATCTTTCTAGGGTTAAAAAAGATTTGCGCTTTACATCGACCTAAACTTTAAAACAGTCATAGAATCTTCGTTTGACAAAAACCACATTGTGGCCAAGCTGTGTGGAATTCGTAATCATGGTCATAGCTGTTTCCTGTGTGAAATTGTTATCCGCTCACAATTCCACACAACATACGAGCCGGAAGCATAAAGTGTAAAGCCTGGGGTGCCTAATGAGTGAGCTAACTCACATTAATTGCGTTGCGCTCACTGCCCGCTTTCCAGTCGGGAAACCTGTCGTGCCAGCTGCATTAATGAATCGGCCAACGCGCGGGGAGAGGCGGTTTGCGTATTGGGCGCTCTTCCGCTTCCTCGCTCACTGACTCGCTGCGCTCGGTCGTTCGGCTGCGGCGAGCGGTATCAGCTCACTCAAAGGCGGTAATACGGTTATCCACAGAATCAGGGGATAACGCAGGAAAGAACATGTGAGCAAAAGGCCAGCAAAAGGCCAGGAACCGTAAAAAGGCCGCGTTGCTGGCGTTTTTCCATAGGCTCCGCCCCCCTGACGAGCATCACAAAAATCGACGCTCAAGTCAGAGGTGGCGAAACCCGACAGGACTATAAAGATACCAGGCGTTTCCCCCTGGAAGCTCCCTCGTGCGCTCTCCTGTTCCGACCCTGCCGCTTACCGGATACCTGTCCGCCTTTCTCCCTTCGGGAAGCGTGGCGCTTTCTCATAGCTCACGCTGTAGGTATCTCAGTTCGGTGTAGGTCGTTCGCTCCAAGCTGGGCTGTGTGCACGAACCCCCCGTTCAGCCCGACCGCTGCGCCTTATCCGGTAACTATCGTCTTGAGTCCAACCCGGTAAGACACGACTTATCGCCACTGGCAGCAGCCACTGGTAACAGGATTAGCAGAGCGAGGTATGTAGGCGGTGCTACAGAGTTCTTGAAGTGGTGGCCTAACTACGGCTACACTAGAAGGACAGTATTTGGTATCTGCGCTCTGCTGAAGCCAGTTACCTTCGGAAAAAGAGTTGGTAGCTCTTGATCCGGCAAACAAACCACCGCTGGTAGCGGTGGTTTTTTTGTTTGCAAGCAGCAGATTACGCGCAGAAAAAAAGGATCTCAAGAAGATCCTTTGATCTTTTCTACGGGGTCTGACGCTCAGTGGAACGAAAACTCACGTTAAGGGATTTTGGTCATGAGATTATCAAAAAGGATCTTCACCTAGATCCTTTTAAATTAAAAATGAAGTTTTAAATCAATCTAAAGTATATATGAGTAAACTTGGTCTGACAGTTACCAATGCTTAATCAGTGAGGCACCTATCTCAGCGATCTGTCTATTTCGTTCATCCATAGTTGCCTGACTCCCCGTCGTGTAGATAACTACGATACGGGAGGGCTTACCATCTGGCCCCAGTGCTGCAATGATACCGCGAGACCCACGCTCACCGGCTCCAGATTTATCAGCAATAAACCAGCCAGCCGGAAGGGCCGAGCGCAGAAGTGGTCCTGCAACTTTATCCGCCTCCATCCAGTCTATTAATTGTTGCCGGGAAGCTAGAGTAAGTAGTTCGCCAGTTAATAGTTTGCGCAACGTTGTTGCCATTGCTACAGGCATCGTGGTGTCACGCTCGTCGTTTGGTATGGCTTCATTCAGCTCCGGTTCCCAACGATCAAGGCGAGTTACATGATCCCCCATGTTGTGCAAAAAAGCGGTTAGCTCCTTCGGTCCTCCGATCGTTGTCAGAAGTAAGTTGGCCGCAGTGTTATCACTCATGGTTATGGCAGCACTGCATAATTCTCTTACTGTCATGCCATCCGTAAGATGCTTTTCTGTGACTGGTGAGTACTCAACCAAGTCATTCTGAGAATAGTGTATGCGGCGACCGAGTTGCTCTTGCCCGGCGTCAATACGGGATAATACCGCGCCACATAGCAGAACTTTAAAAGTGCTCATCATTGGAAAACGTTCTTCGGGGCGAAAACTCTCAAGGATCTTACCGCTGTTGAGATCCAGTTCGATGTAACCCACTCGTGCACCCAACTGATCTTCAGCATCTTTTACTTTCACCAGCGTTTCTGGGTGAGCAAAAACAGGAAGGCAAAATGCCGCAAAAAAGGGAATAAGGGCGACACGGAAATGTTGAATACTCATACTCTTCCTTTTTCAATATTATTGAAGCATTTATCAGGGTTATTGTCTCATGAGCGGATACATATTTGAATGTATTTAGAAAAATAAACAAATAGGGGTTCCGCGCACATTTCCCCGAAAAGTGCCACCTGACGTCTAAGAAACCATTATTATCATGACATTAACCTATAAAAATAGGCGTATCACGAGGCCCTTTCGTCTCGCGCGTTTCGGTGATGACGGTGAAAACCTCTGACACATGCAGCTCCCGGAGACGGTCACAGCTTGTCTGTAAGCGGATGCCGGGAGCAGACAAGCCCGTCAGGGCGCGTCAGCGGGTGTTGGCGGGTGTCGGGGCTGGCTTAACTATGCGGCATCAGAGCAGATTGTACTGAGAGTGCACCATATGCGGTGTGAAATACCGCACAGATGCGTAAGGAGAAAATACCGCATCAGGCGCCATTCGCCATTCAGGCTGCGCAACTGTTGGGAAGGGCGATCGGTGCGGGCCTCTTCGCTATTACGCCAGCTGGCGAAAGGGGGATGTGCTGCAAGGCGATTAAGTTGGGTAACGCCAGGGTTTTCCCAGTCACGACGTTGTAAAACGACGGCCAGTGCCAAGCTTTGTTTAAAAATATAACAAAATTGTGATCCCACAAAATAAAGTGGGGCAAAATTAAATAATTAATAGTGTCTGTAAACTTGTTGGTCTTCAACTTTTTGAGAAACACGTTGGACGGCAAATCTGTGACTATAACACAAGTTGATTTGATAATTTTAGCCAACACGTCGGGCTGCGTGTTTTTCACCGACGCGTCTGTGTACACGTTGATTAATTGGTCGATTAAACTGTTGAAATAATTTAATTTTTGGTTCTTCTTTAAATCTGTGATGAAATTTTTTAAAATAACTTTAAATTCTTCATTGGTAAAAAATGCCACGTTTTGCAACTTGTGAGGGTCTAATATGAGGTCAAACTCAGTAGGAGTTTTATCCAAAAAAGAAAACATGATTACGTCTGTACACGAACGCGTATTAACGCAGAGTGCAAAGTATAAGAGGGTTAAAAAATATATTTTACGCACCATATACGCATCGGGTTGATATAGTTAATATGGATCAATTTGAACAGTTGATTAACGTGTCTCTGCTCAAGTCGTTGATCAAAACGCAAATCGACGAAAATGTGTCGGACAATATCAAGTCGATGAGCGAAAAACTAAAAAGGCTAGAATACGACAATCTCACAGACAGCGTTGAGATATACGGTATTCACGACAGCAGGCTGAATAATAAAAAAATTAGAAACTATTATTTAACCCTAGAAAGATAATCATATTGTGACGTACGTTAAAGATAATCATGCGTAAAATTGACGCATGTGTTTTTATCGGTCTGTATATCGAGGTTTATTTATTAATTTGAATAGATATTAAGTTTTATTATATTTACACTTACATACTAATAATAAATTCAACAAACAATTTATTTATGTTTATTTATTTATTAAAAAAAAACAAAAACTCAAAATTTCTTCTAAAGTAACAAAACTTTA

Note: the sequences marked green color: the coding sequences of the Human serum albumin; the sequences marked red color: the sequences of the recognition site of enterokinase , DDDDK; the sequences marked purple color: the sequences of His6 tag; the sequences marked blue color: the sequences of the signal peptide of the *fibroin light chain gene;* the sequences marked orange color: the sequences of the promoter sequence of the *fibroin light chain gene;* the sequences marked light blue color: the coding sequences of the polyA signal sequence of the *fibroin light chain gene.*
