# Supplementary figures and images for: High-efficiency production of human serum albumin in the posterior silk glands of transgenic silkworms, Bombyx mori L
Source: PLoS One. 2018 Jan 19;13(1):e0191507. doi: 10.1371/journal.pone.0191507 (PMC5774803; doi:10.1371/journal.pone.0191507)

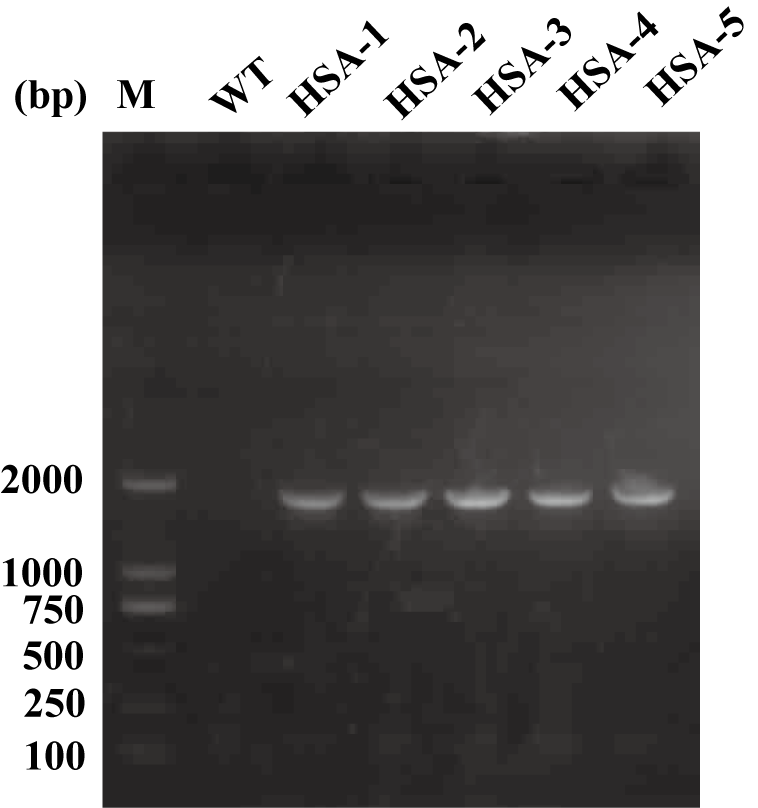

Supplement: S1 Fig — (TIF) [file pone.0191507.s002.tif]
