# Supplementary material for: High-efficiency production of human serum albumin in the posterior silk glands of transgenic silkworms, Bombyx mori L
Source: PLoS One. 2018 Jan 19;13(1):e0191507. doi: 10.1371/journal.pone.0191507 (PMC5774803; doi:10.1371/journal.pone.0191507)
Supplement: S2 Table — (DOCX) [file pone.0191507.s004.docx]

**S2. Table The content of rHSA protein in the cocoon shells from the HSA-2 transgenic silkworm pedigree.**

|  | HSA-2(1) | HSA-2(2) | HSA-2(3) | Average |  |
| --- | --- | --- | --- | --- | --- |
| Percentage of rHSA protein in the soluble proteins (%) | 35.19 % | 29.92% | 22.12% | 29.07% |  |
| Percentage of rHSA protein in the cocoon sample (%) | 2.11% | 1.79% | 1.33 % | 1.74% |  |

Note: Each 10-mg cocoon sample was suspended in 100 μl PBS solution and ground with a homogenizer into powder on ice.
